# Extended Line Map-Based Precise Vehicle Localization Using 3D LIDAR

**DOI:** 10.3390/s18103179

**Published:** 2018-09-20

**Authors:** Jun-Hyuck Im, Sung-Hyuck Im, Gyu-In Jee

**Affiliations:** 1 Department of Electronic Engineering, Konkuk University, 120 Neungdong-ro, Gwangjin-gu, Seoul 05029, Korea; junhyuck@konkuk.ac.kr; 2 Satellite Navigation Team, Korea Aerospace Research Institute, 169-84 Gwahak-ro, Yuseong-gu, Daejeon 305-806, Korea; ish@kari.re.kr

**Keywords:** extended line map, precise vehicle localization, 3D LIDAR, road marking, vertical structure

## Abstract

An Extended Line Map (ELM)-based precise vehicle localization method is proposed in this paper, and is implemented using 3D Light Detection and Ranging (LIDAR). A binary occupancy grid map in which grids for road marking or vertical structures have a value of 1 and the rest have a value of 0 was created using the reflectivity and distance data of the 3D LIDAR. From the map, lines were detected using a Hough transform. After the detected lines were converted into the node and link forms, they were stored as a map. This map is called an extended line map, of which data size is extremely small (134 KB/km). The ELM-based localization is performed through correlation matching. The ELM is converted back into an occupancy grid map and matched to the map generated using the current 3D LIDAR. In this instance, a Fast Fourier Transform (FFT) was applied as the correlation matching method, and the matching time was approximately 78 ms (based on MATLAB). The experiment was carried out in the Gangnam area of Seoul, South Korea. The traveling distance was approximately 4.2 km, and the maximum traveling speed was approximately 80 km/h. As a result of localization, the root mean square (RMS) position errors for the lateral and longitudinal directions were 0.136 m and 0.223 m, respectively.

## 1. Introduction

Currently, precise vehicle localization is being recognized as a key technology for autonomous driving. Although there are no standards for the localization accuracy required for autonomous driving, the report in [1] requires a localization accuracy of less than 0.7 m in the lateral direction at a 95% confidence level. Recently, a localization accuracy of less than 0.5 m in the lateral direction and less than 1 m in the longitudinal direction has been generally required at a 95% confidence level. In general, the accuracy of the Real-Time Kinematic (RTK) Global Positioning System (GPS) meets such requirements. In urban areas, however, even the costly RTK GPS/Inertial Measurement Unit (IMU) integrated system cannot meet the localization accuracy requirements for autonomous driving.

To address this problem, many studies are being conducted to improve localization accuracy through the convergence of various sensors mounted on autonomous vehicles. In particular, 3D Light Detection and Ranging (LIDAR) is being used as a key sensor for precise vehicle localization. As LIDAR can provide accurate distances and reflectivity information of surrounding objects, it can also estimate the relative vehicle position for surrounding objects in a very accurate manner. Therefore, precise vehicle localization is possible using 3D LIDAR if precision maps for the surroundings are available.

Precision maps are essential for precise vehicle localization. In general, precision maps for LIDAR-based precise vehicle localization are divided into point maps, line maps, 2D/2.5D plane maps, and 3D maps.

First, there are the most basic localization methods based on point maps. In the papers of [2] and [3], the line components of a building wall were extracted from LIDAR data, and both end-points of the lines or corners where two lines met were detected and used for localization. In these papers, however, the quantitative localization accuracy was not derived, and only the point landmark detection performance was analyzed. The study in [4] used columns such as street trees and lamps as point landmarks based on their characteristics perpendicular to the ground. However, the localization produced position errors higher than 0.5 m in many areas. The study in [5] detected the vertical corners of buildings and used them for precise vehicle localization. As such, vertical corners are perpendicular to the ground and can be expressed as point landmarks on a 2D horizontal plane. For this reason, the vertical corner map had a very small data size (28 KB). However, vertical corners were not detected in some areas, and in such areas, an increase in the vehicle position error is inevitable. Furthermore, this method cannot be used in areas that have no buildings.

Secondly, there are localization methods that use line detection. In the papers of [6,7], the horizontal line components of a hallway were detected using a LIDAR mounted on an indoor traveling robot, and localization was performed through the distance and angle information between the lines and the robot. In an indoor hallway, it is possible to easily detect the line components of the wall. Outdoors, however, line detection is not easy as the surrounding buildings and structures have extremely complicated shapes and are hidden by obstacles, such as street trees. For autonomous vehicle localization, road markings such as lanes are typically used. Such road markings can be detected using the reflectivity information of LIDAR and are used to create a line map [8]. In addition, it is possible to correct the position error of a vehicle by matching the detected lane and the line map [9]. For the study in [9], the curb was extracted using LIDAR. From the curb, the area of interest was set and lanes were detected. As a result of localization, the average errors in the lateral and longitudinal directions were 0.14 and 0.20 m, respectively. However, position errors higher than 0.5 m occurred in many sections.

Thirdly, there are localization methods that use plane maps. Hybrid maps were generated by integrating the lane and landmark information with 2D occupancy grid maps, and localization was performed using the hybrid maps [10,11,12]. In the paper of [13], a multilevel surface map was used for localization in a multi-floor parking lot. Studies in which 2D LIDAR data were accumulated on a plane and applied to localization were also conducted [14,15]. In the papers of [16,17], a road reflectivity map was generated and used to perform localization. However, the road reflectivity map was significantly affected by changes in weather and illumination, as well as the presence of vehicles.

To deal with such shortcomings, methods using vertical structures were researched [18,19]. A multiresolution Gaussian mixture map in which the height information of vertical structures is stored in a 2D grid map was proposed. The localization using this method produced RMS position errors of 10 and 13 cm in the lateral and longitudinal directions, respectively. Despite the excellent localization performance, the multiresolution Gaussian mixture map had a very large data size (44.3 MB/km). Most of the localization methods using plane maps require extremely large calculation amounts for map matching.

Finally, there are localization methods based on 3D maps. Recently, localization methods using Normal Distribution Transformation (NDT) scan matching have been researched [20,21]. The NDT scan matching exhibits relatively accurate results with a standard deviation of the horizontal position error of less than approximately 30 cm. However, the calculation time is very long (approximately 2.5 s).

In general, the localization accuracy and reliability increases alongside the amount of information. On the other hand, the data file size increases along with the calculation amount for map matching. For real-time vehicle localization, the data file size and calculation amount must be small. Therefore, it is important to ensure the highest localization accuracy and reliability using a map with a small amount of information.

As lanes must exist on roads where vehicles travel, map production companies are producing lane maps most preferentially. Furthermore, such lane maps are stored in line form to minimize the data file size. However, methods with high localization accuracy and reliability among the existing localization methods using LIDAR mostly use maps with very large data sizes. Therefore, research to ensure high localization accuracy using actual produced line maps is necessary. Consequently, considering the compatibility with actual produced maps as well as the data size, it is deemed most effective that maps for LIDAR-based localization also have a line form.

In this paper, a road reflectivity map and occupancy grid map for surrounding vertical structures were generated, and line components were extracted from each map. The extracted lines were stored in a map in node and line forms. This map is called an extended line map (ELM) in this paper. As an ELM includes all information for road markings and vertical structures, it can mutually supplement the shortcomings of both, and thus can ensure high localization accuracy, reliability, and availability.

**Figure 1:**
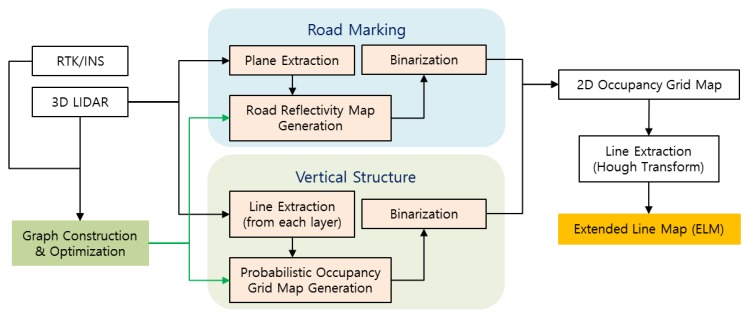
Extended line map (ELM) generation process.

Although the road marking information is stored in ELM in line form, the type of road marking to which each line belongs is not expressed. Likewise, for vertical structures, it is not expressed whether each line belongs to a building or a traffic sign. In the case of ELM-based localization, the ELM is converted into an occupancy grid map and is used for correlation matching. For this reason, whether the lines included in ELM are actual lines is not important, but it is important that certain road markings or vertical structures including lines are present in the area. As a result, as no lines can be detected from areas where nothing exists, it is not necessary to verify whether the lines were properly detected when generating an ELM map. Figure 1 describes the generation process of an ELM (refer to Section 2).

**Figure 2:**
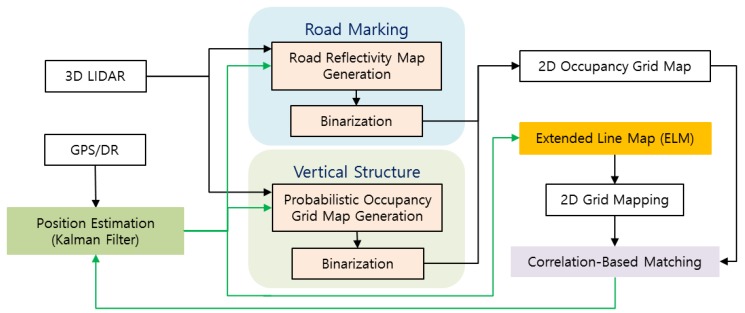
Localization process using ELM.

When localization is performed using an ELM generated through the process shown in Figure 1, the ELM is converted back into an occupancy grid map, and correlation matching with the generated occupancy grid map is performed using the currently acquired LIDAR data. This matching result is used as the measurement of a Kalman Filter (KF). Figure 2 describes the localization process using an ELM (refer to Section 3).

**Figure 3:**
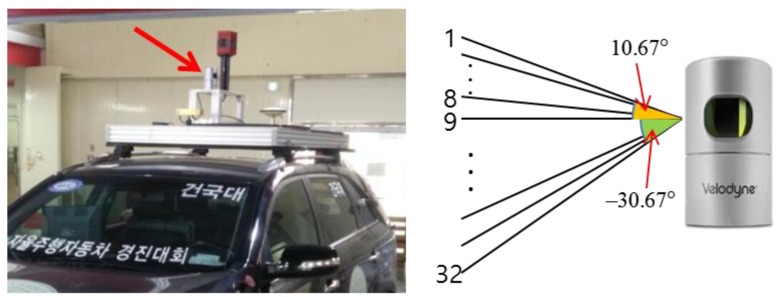
(**Left**) Installation position of Velodyne HDL-32E and (**Right**) vertical layer specifications.

The 3D LIDAR sensor used in this paper was a Velodyne HDL-32E, which was installed on top of a vehicle, as shown in Figure 3. In addition, layers 1 through 16 were used to generate an occupancy grid map for vertical structures, and layers 17 through 32 were used to generate a road reflectivity map.

The proposed ELM has the following benefits:

- It includes all information for road markings and vertical structures.
- It has a very small data file size (approximately 134 KB/km).
- It can be generated through a map generation algorithm, and no verification procedure is required.
- It is compatible with actual produced line maps for lanes.
- In addition, the proposed ELM-based localization method has the following benefits:
- It meets the localization accuracy requirements for autonomous driving.
- As it is used after being converted into an occupancy grid map, line detection and data association processes are not required.
- A Fast Fourier Transform (FFT) can be applied to the correlation matching of the binary occupancy grid map, and the correlation matching time is very short (78 ms on average).

Section 2 describes how to generate an ELM, and Section 3 explains the ELM-based localization method. Section 4 analyzes the ELM-based localization performance, and Section 5 concludes this paper.

## 2. How to Generate an ELM

As described in the paper of [17], when vehicle localization is performed through a road reflectivity map and 2D plane matching, it is not necessary to know what sign a certain road marking represents. In other words, it is not necessary that the map has all of the shape information. This means that road markings can be modeled in simple forms and applied to localization when the 2D plane-matching method is used.

All roads where vehicles travel basically have lanes drawn, and also include many additional road markings, such as stop lines, crosswalks, and arrows. Maps of roads which are currently being produced necessarily include information on lanes, which is typically in the form of lines. Such lane maps have already been applied to vehicle localization [8,9]. However, as most road markings other than lanes also have similar forms to lines, they can all just be expressed as a set of lines. Therefore, line maps with the same form as existing lane maps can be generated. They can be converted into 2D plane maps and applied to vehicle localization.

It is difficult to use road markings in areas with traffic congestion. In urban areas, many tall buildings are present around roads, and they can always be scanned regardless of traffic congestion. Therefore, it is necessary to use buildings to increase localization availability. Furthermore, the outer walls of urban buildings are mostly composed of planes and are perpendicular to the ground. For this reason, such outer walls are mostly expressed as lines when a 2D occupancy grip map is generated using 3D LIDAR. Such lines can be expressed in the same form as the lines extracted from road markings. As seen, lines are extracted from the reflectivity map for the road surface and the occupancy grid map for buildings, and the extracted lines are converted into node and line forms. Finally, the position of each node is stored in the ELM. Next, the method for ELM generation is explained.

### 2.1. Vehicle Trajectory Optimization

**Figure 4:**
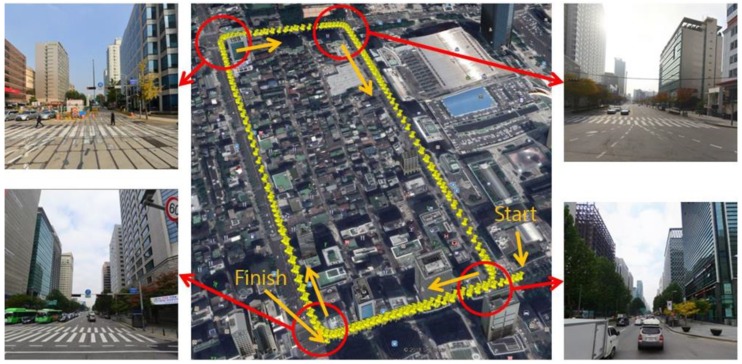
Vehicle trajectory and street view (four intersections).

An experiment was carried out in the Gangnam area of Seoul, South Korea. Figure 4 shows the vehicle trajectory and the street view at the four intersections.

**Figure 5:**
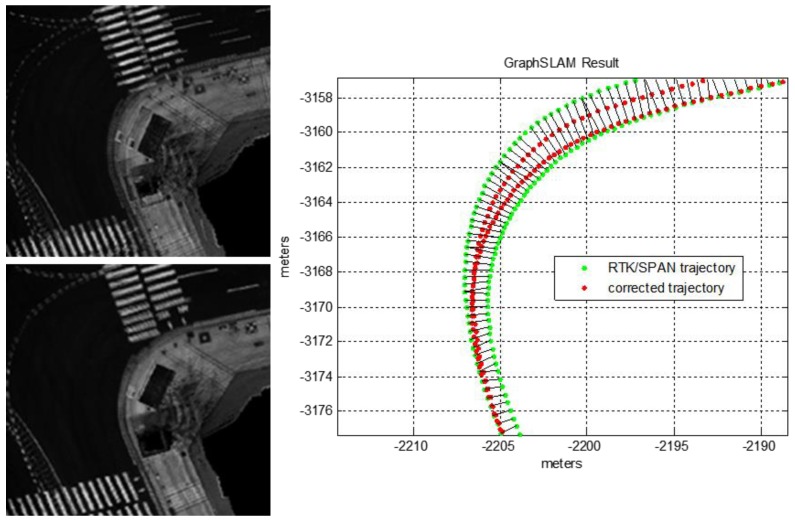
Graph optimization result of vehicle trajectory using GraphSLAM.

As shown in Figure 4, the experimental environment is a dense urban area surrounded by tall buildings, and two laps were driven from the starting point to the finishing point. The traveling distance was approximately 4.2 km, and the maximum traveling speed and average speed were approximately 80 km/h and 32 km/h, respectively. The position of the vehicle was acquired by using the integrated system of the RTK GPS and Inertial Navigation System (INS) (NovAtel RTK/SPAN system). In an environment where there are many tall buildings, the position error of the RTK/INS is about 1–2 m. Therefore, the vehicle trajectory must be corrected to generate the precision map. The vehicle trajectory was optimized by using the GraphSLAM method. Figure 5 shows the graph optimization result of the vehicle trajectory.

As shown at the top left of Figure 5, the road reflectivity maps for the two laps do not match. On the right of Figure 5, the red points represent the corrected vehicle trajectory after the graph optimization. As shown at the bottom left of Figure 5, the road reflectivity map matches exactly. Here, the incremental pose information outputted from the Iterative Closest Point (ICP) algorithm was used as an edge measurement of the graph. The theory and principles for the GraphSLAM method are well described in [22,23]. Thus, obtaining the optimized vehicle trajectory is possible by using the GraphSLAM. In this paper, this optimized vehicle trajectory was considered as the ground truth.

### 2.2. Line Extraction from Road Reflectivity Map

**Figure 6:**
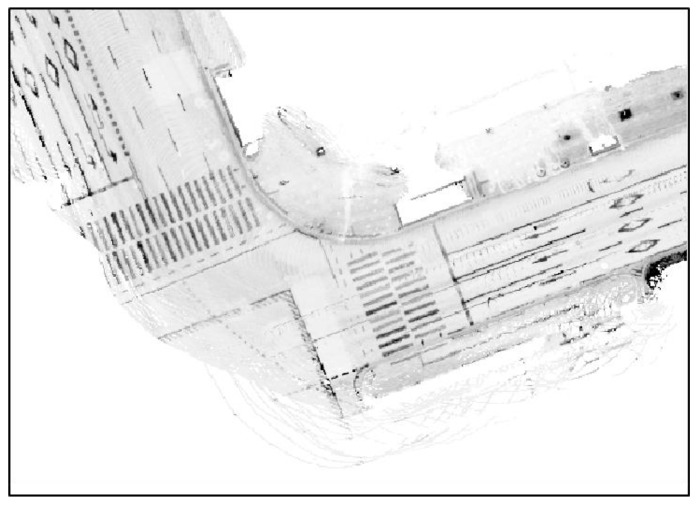
Generated road reflectivity map (before plane extraction).

First, a road reflectivity map with a grid size of 15 cm was generated using the vehicle position optimized in Section 2.1. Figure 6 shows the generated road reflectivity map.

**Figure 7:**
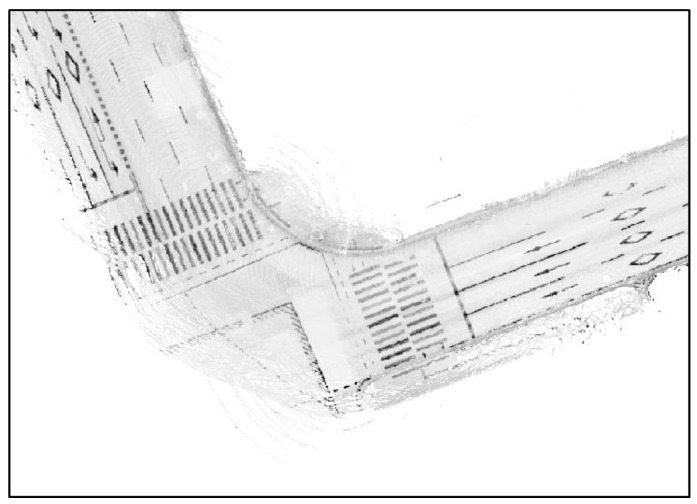
Generated road reflectivity map (after plane extraction).

The reflectivity map of Figure 6 was generated using layers 17 through 32 of the 3D LIDAR. As seen in the figure, the reflectivity map includes the reflected parts of sidewalks or nearby vehicles. These parts must be eliminated as they may degrade the localization performance. Plane extraction was performed to extract only the LIDAR points reflected from the road. Figure 7 shows the road reflectivity map generated after plane extraction. 

**Figure 8:**
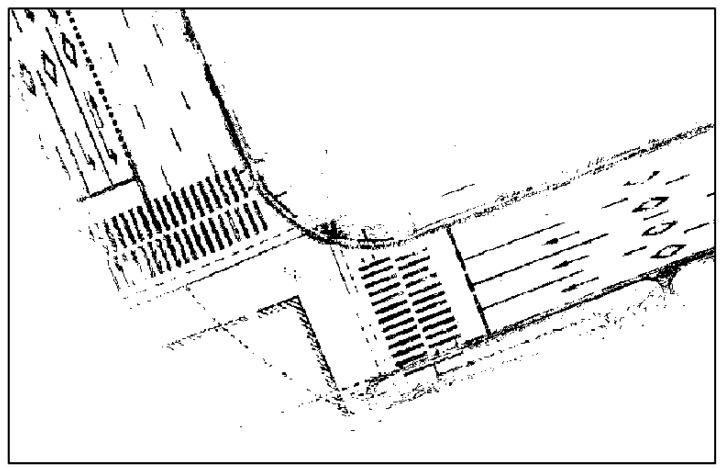
Binarized road reflectivity map.

In Figure 7, the sidewalks have not been completely eliminated, but most of the unnecessary parts have been removed. Now, lines must be extracted from the reflectivity map, as shown in Figure 7. In the reflectivity map, however, areas except for road markings are filled with certain reflectivity values. As lines can be extracted from these areas, it is necessary to eliminate the values of such areas. In general, the reflectivity value differs greatly between road markings and other areas. Therefore, the values of such areas can be eliminated in a simple manner through binarization. In this paper, binarization was performed using the Otsu thresholding method [24]. Figure 8 shows the results of applying binarization to the reflectivity map of Figure 7.

**Figure 9:**
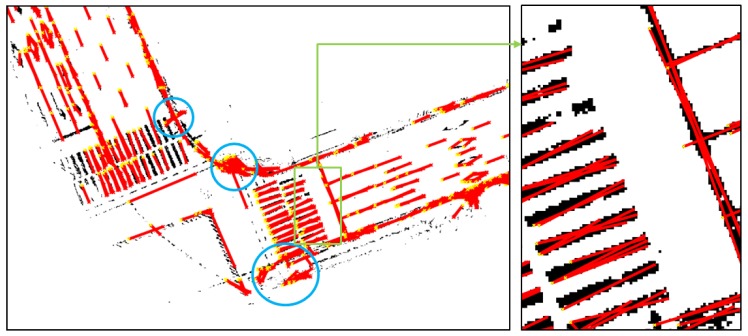
Line extraction results for road markings.

As shown in Figure 8, some parts of the sidewalks and curbs have not been eliminated, but most of the road markings remain. Now, lines are extracted from the binary map, as shown in Figure 8. A Hough transform was used as the line extraction algorithm. Figure 9 shows the line extraction results for the road markings. 

In Figure 9, it can be seen that lines have been extracted from most of the road markings. As can be seen from the right-hand figure, multiple lines have been extracted from thick road markings. Therefore, the actual information on the shape can be retained as much as possible. However, as the parts marked with blue circles show, lines have been extracted from the parts that are not road markings. It is difficult to eliminate these parts because they have the same reflectivity characteristics as road markings even though they are not road markings. However, the incorrectly detected lines do not significantly affect the correlation matching results because many clear road markings are present nearby.

### 2.3. Line Extraction from Occupancy Grid Map

**Figure 10:**
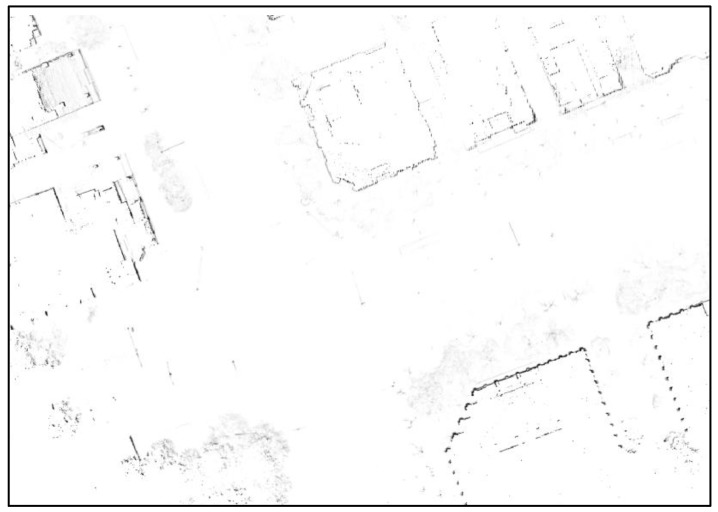
Generated 2D probabilistic occupancy grid map.

This paper uses the fact that the outer walls of buildings are expressed as lines on the 2D horizontal plane. To extract highly reliable line information, a 2D probabilistic occupancy grid map for vertical structures was generated, and lines were extracted from the map. Figure 10 shows the generated 2D probabilistic occupancy grid map.

As can be seen from Figure 10, the outer walls of buildings appear as lines on the 2D plane map. However, the occupancy probability for the outer walls of the buildings is low owing to the influence of the street trees or building forms, and many street trees around the road remain on the map. As only lines are required on the map to be used for localization, it is necessary to eliminate unnecessary parts as much as possible. For this, line components are extracted from the LIDAR point cloud, and a probabilistic occupancy grid map is generated using only the extracted points. In this case, street trees can be effectively removed from the probabilistic occupancy grid map, and the occupancy probability for the building outer walls can be increased. 

**Figure 11:**
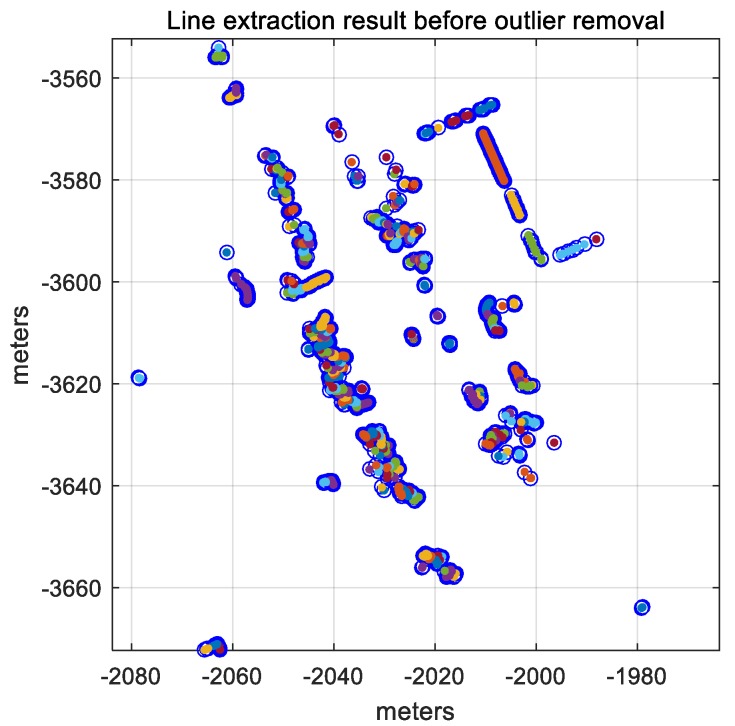
Line extraction result using the IEPF algorithm.

There are several methods to extract lines from the LIDAR point cloud [2,25,26,27]. Among the methods, the Iterative-End-Point-Fit (IEPF) algorithm exhibits the best performance in terms of accuracy and calculation time [28]. Figure 11 shows the results of line extraction using the IEPF algorithm.

**Figure 12:**
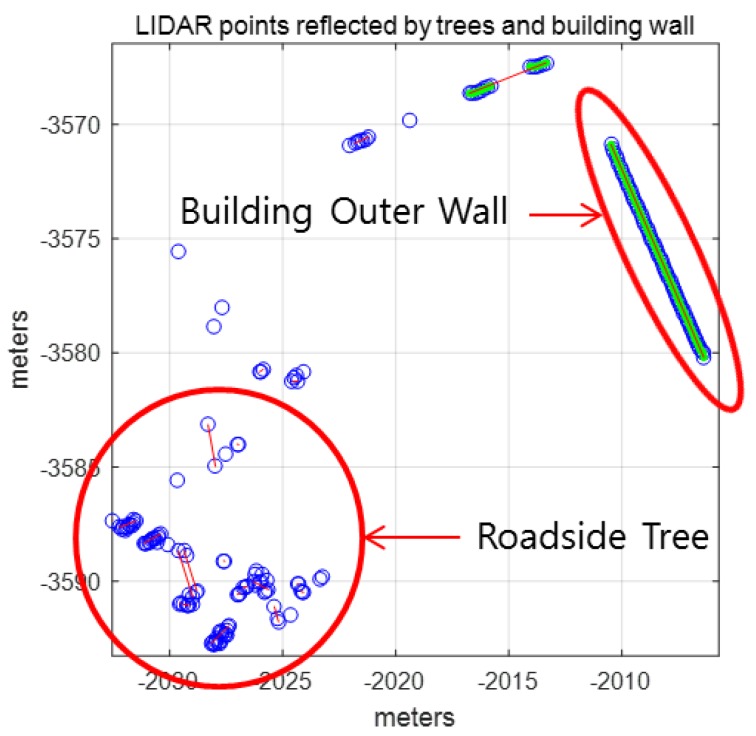
Light Detection and Ranging (LIDAR) points reflected by leaves of roadside trees and outer wall of building.

In Figure 11, most of these line segments are a set of scan points from the leaves of the roadside trees. Therefore, the laser data reflected by the leaves of roadside trees must be removed. Figure 12 shows the LIDAR points reflected by the leaves and the outer wall of the building.

**Figure 13:**
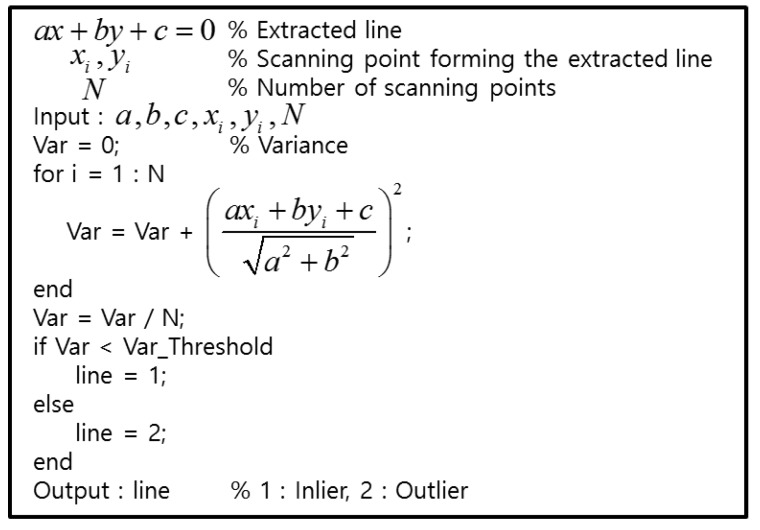
Pseudocode for outlier removal.

As shown in Figure 12, the features of the LIDAR points that are reflected by the two types of objects are clearly distinguished. For roadside trees, the variance of distance errors between the extracted line and each point is very large. On the other hand, for the outer wall of a building, the variance of the distance errors is very small. Figure 13 shows the pseudocode for outlier removal.

**Figure 14:**
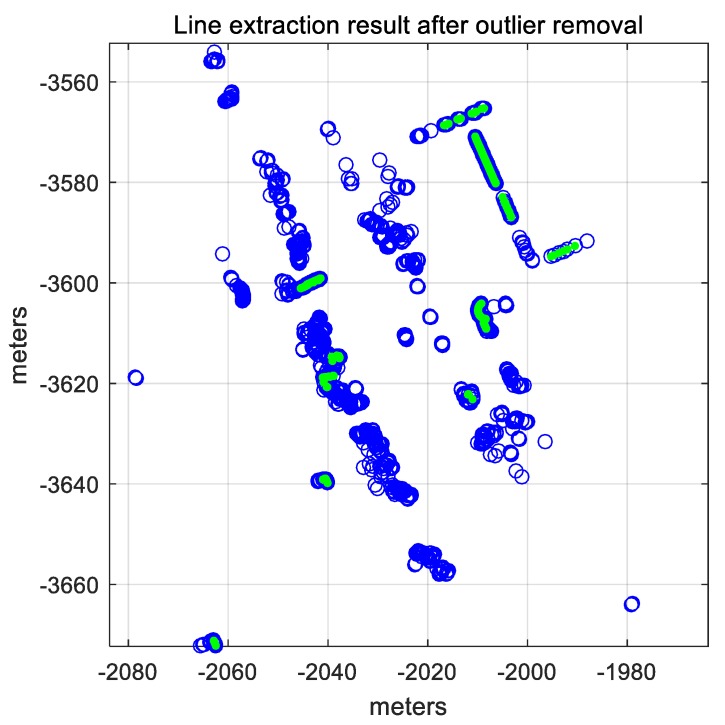
Line extraction result from LIDAR points (after outlier removal).

By using the pseudocode, outliers such as roadside trees can be removed. Figure 14 shows the line extraction result after the outlier removal.

**Figure 15:**
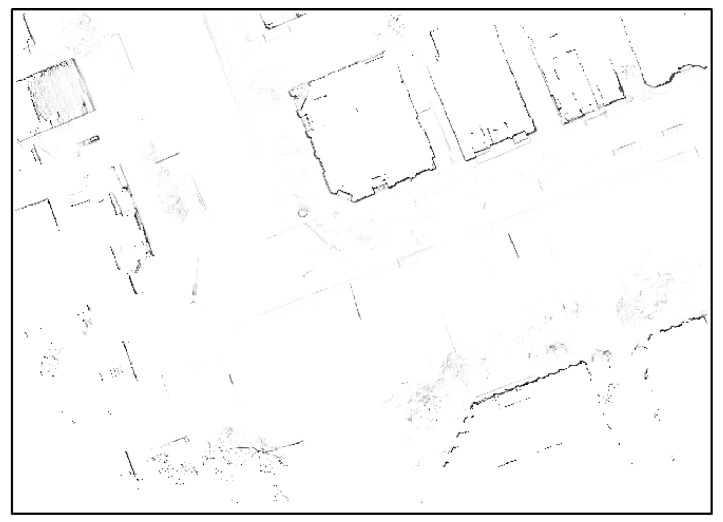
Probabilistic occupancy grid map generated by applying the IEPF algorithm.

In Figure 14, the green points represent the line segments that are finally extracted. The probabilistic occupancy grid map is generated again using only the LIDAR points corresponding to the extracted lines. Figure 15 shows the probabilistic occupancy grid map generated by applying the IEPF algorithm.

**Figure 16:**
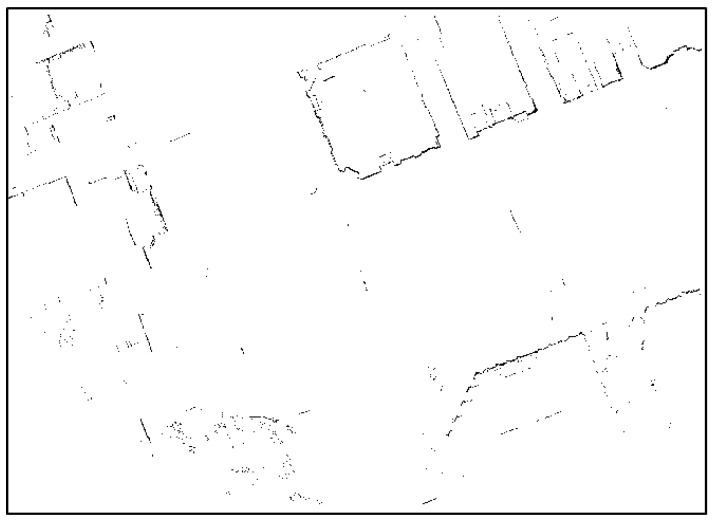
Generated occupancy grid map.

As can be seen from Figure 15, many street trees have been eliminated, but some of them remain. Furthermore, the vertical structures reflecting only some layers have low probability values. However, it can be seen that the probability values for the building outer walls are higher in Figure 15 than in Figure 10. This is because only lines with relatively high reliability have been mapped to the map through the IEPF algorithm. Binarization is performed to remove grids with low probabilities. A value between 0 and 1 is stored in each grid. This paper assumes that grids with values equal to or higher than 0.5 were occupied. Figure 16 shows the final generated occupancy grid map after binarization.

**Figure 17:**
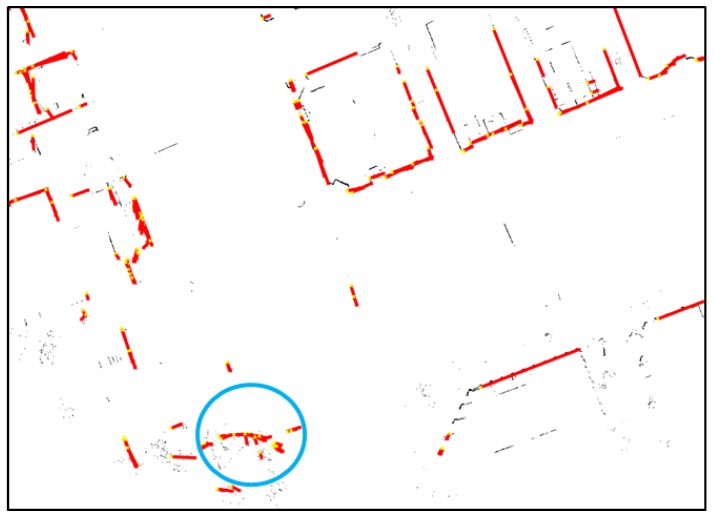
Line extraction result for vertical structures (incorrectly detected lines in a blue circle).

Figure 16 shows that most street trees have been removed and only the outer walls of buildings or traffic signs remain. Next, lines were extracted from this occupancy grid map. As with Section 2.2, a Hough transform is used as the line extraction algorithm. Figure 17 shows the final line extraction result.

In Figure 17, lines have been extracted from parts other than buildings as areas marked with a blue circle. However, as with the case of road markings, incorrectly detected lines do not significantly affect correlation matching because many other vertical structures are present nearby. In this way, lines for vertical structures can be extracted.

### 2.4. Generation of ELM

The lines extracted in Section 2.2 and Section 2.3 were converted into nodes and links, and stored in a map. Table 1 presents an example of the ELM.

**Figure 18:**
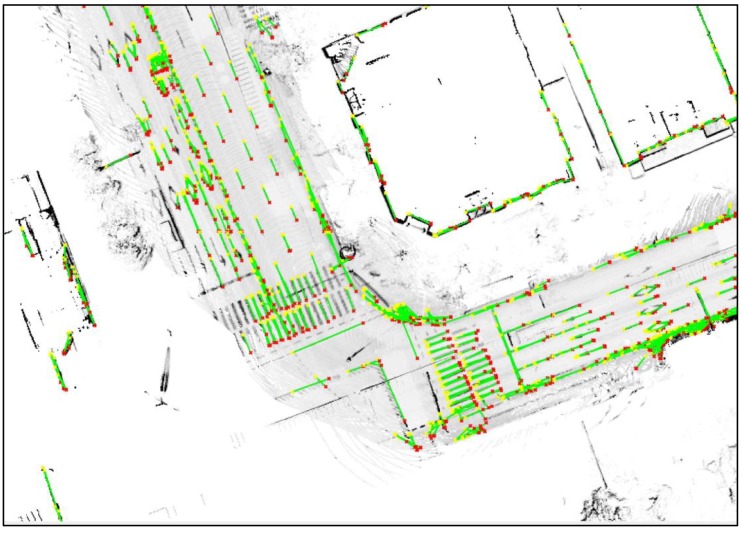
Generated ELM (on synthetic map).

As shown in Table 1, the position information of each line was stored in a text file. The data size of the created ELM was approximately 134 KB/km. The ELM has a significantly smaller data size than other maps used for 3D LIDAR-based localization. Figure 18 shows the ELM information displayed on a synthetic map of the 2D occupancy grid and road reflectivity maps.

In Figure 18, the yellow star and red star represent the positions of node 1 and node 2, respectively. The green line connects node 1 and node 2. In this study, localization was performed using an ELM created in this way. 

## 3. ELM-Based Localization Method

### 3.1. Correlation-Based Matching Using ELM

**Figure 19:**
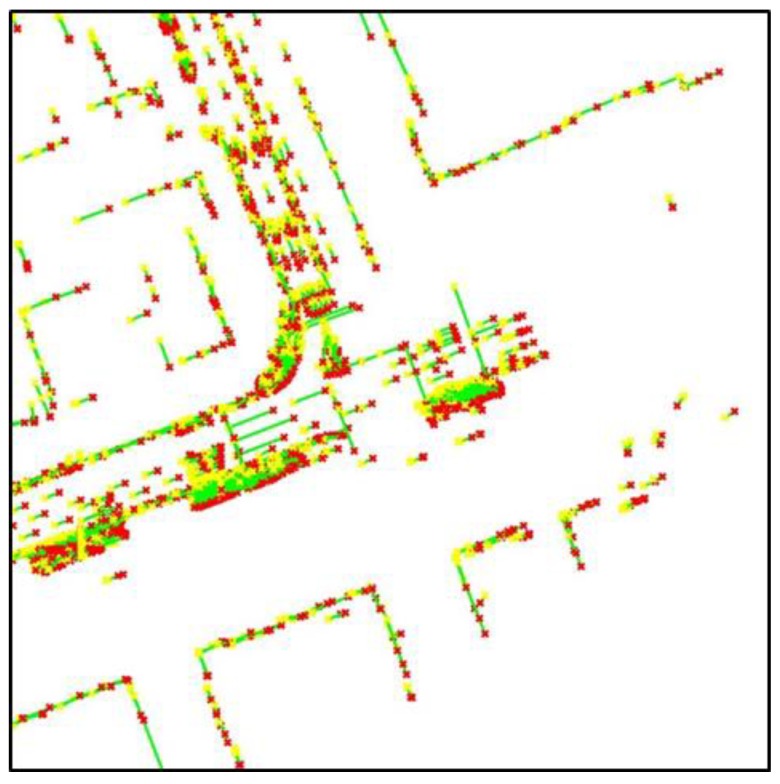
ELM in area of interest.

**Figure 20:**
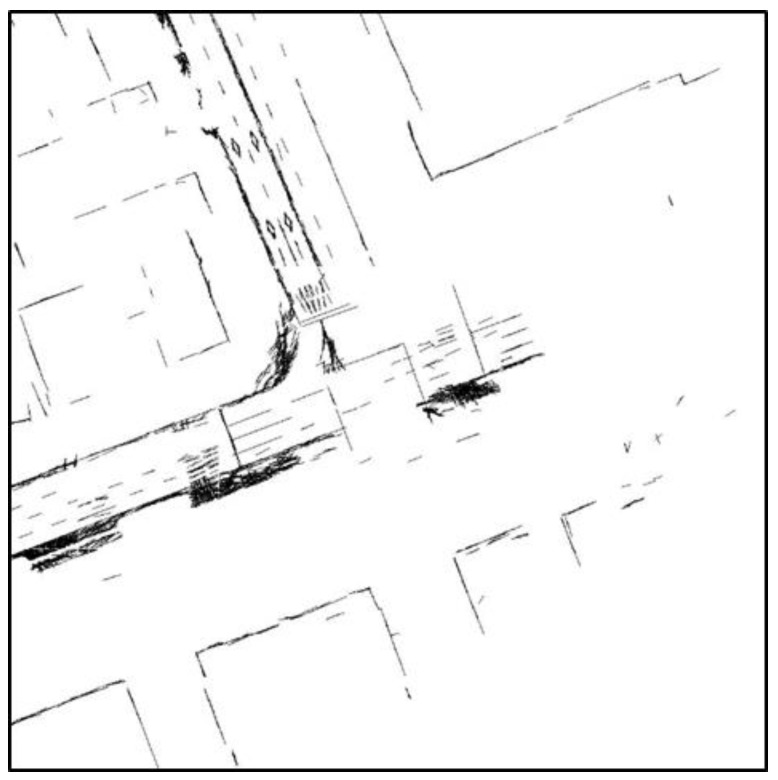
Result of converting Figure 19 into occupancy grid map.

As shown in Figure 2, localization was performed through correlation matching of the occupancy grid map converted from ELM and the occupancy grid map generated using the current LIDAR data. First, nodes present within a certain distance from the current vehicle position were selected from the ELM. The current position information is acquired from a commercial GPS/Dead Reckoning (DR) sensor. Figure 19 shows the node and link information of the ELM present in the area of interest on the 2D plane. Furthermore, the lines in Figure 19 can be mapped to the grid map. Figure 20 shows the result of converting the line map into an occupancy grid map.

**Figure 21:**
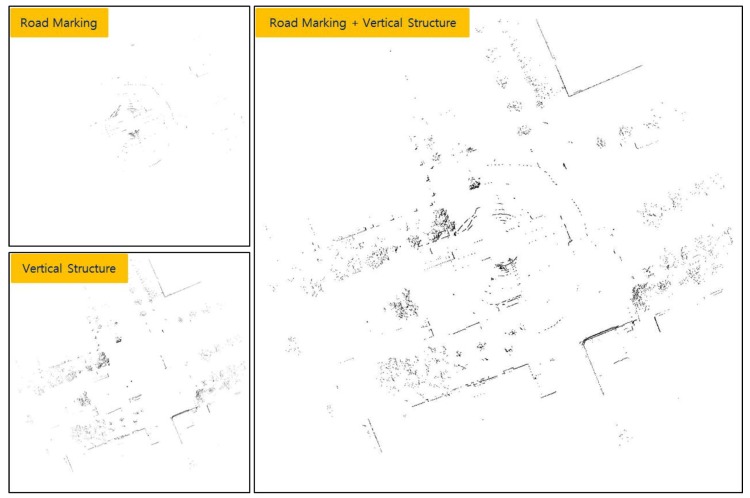
Binary occupancy grid map generated using current LIDAR data.

Next, an occupancy grid map with the same size as the map of Figure 20 was generated using the current LIDAR data. First, a road reflectivity map was generated and binarized in the same way as the case of ELM generation. Then, a probabilistic occupancy grid map for vertical structures was generated, and an occupancy grid map was generated through binarization. The two generated maps were composed of 0 and 1. Therefore, the two maps were integrated to complete an occupied grid map. Figure 21 shows the generated binary occupancy grid map.

**Figure 22:**
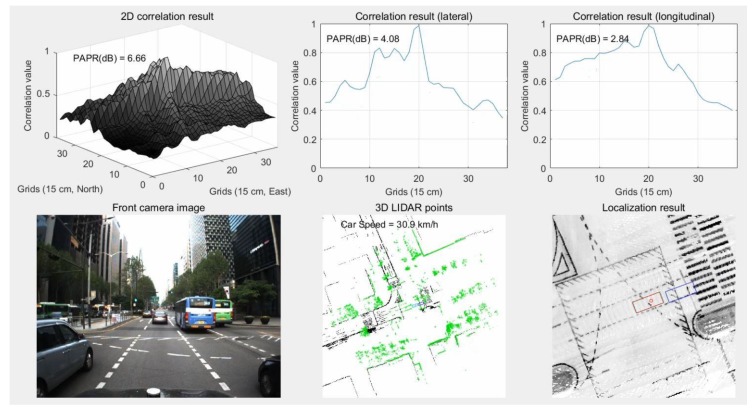
Localization execution process.

Subsequently, correlation matching was performed for the two occupancy grid maps of Figure 20 and Figure 21. In this paper, an area of 160 m × 160 m was set as the area of interest, and the grid size was 15 cm. The maps used for matching became 1081 × 1081 matrices. Therefore, it was difficult to use the general serial-search-type correlation matching method. However, as the two occupancy grid maps were composed of only 0 and 1, the matching of the two maps could simply be calculated through multiplying the two matrices. Consequently, it was possible to apply an FFT. When correlation matching is performed using an FFT, the calculation time is approximately 78 ms (based on MATLAB). The time required for coordinate transformation of the 3D LIDAR point cloud and generation of the binary occupancy grid map is approximately 94 ms and 142 ms (based on MATLAB), respectively. Therefore, the localization execution time is less than 350 ms. In this paper, localization was performed by post-processing to verify the performance using all LIDAR data (10 Hz). Figure 22 shows the localization execution process.

As shown in Figure 22, experiments were performed in a downtown area where many other vehicles were present. In the bottom center of the figure, the light green dots represent LIDAR scan data to be matched with the map. After these dots and the ELM in the area of interest were converted into binary occupancy grid maps, the results of the correlation matching (FFT) between the two maps were used for the measurements of the KF. In the bottom right of the figure, the red rectangle represents the currently estimated vehicle position, while the black rectangle represents the actual position (ground truth). The blue and light blue rectangles represent the positions of GPS/DR and RTK/INS, respectively. The fact that the red and black rectangles almost overlap indicates that the position of the vehicle was estimated accurately. 

### 3.2. Kalman Filter Configuration

**Figure 23:**
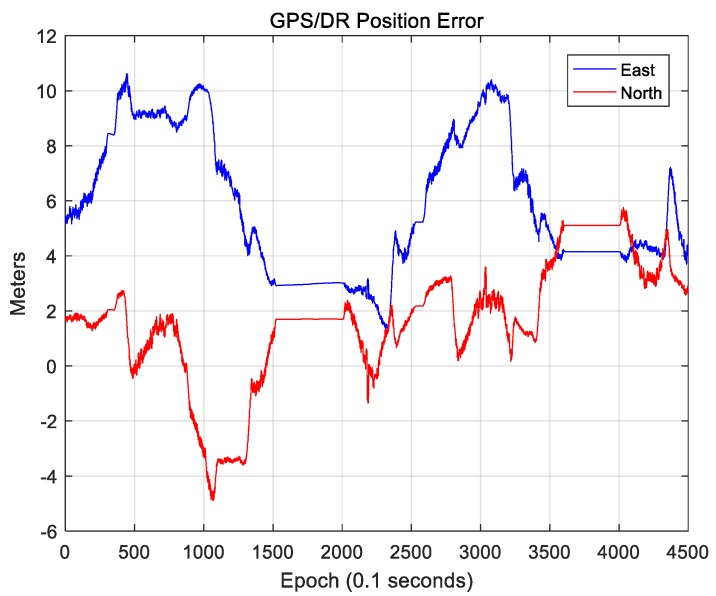
Position error of GPS/DR.

**Figure 24:**
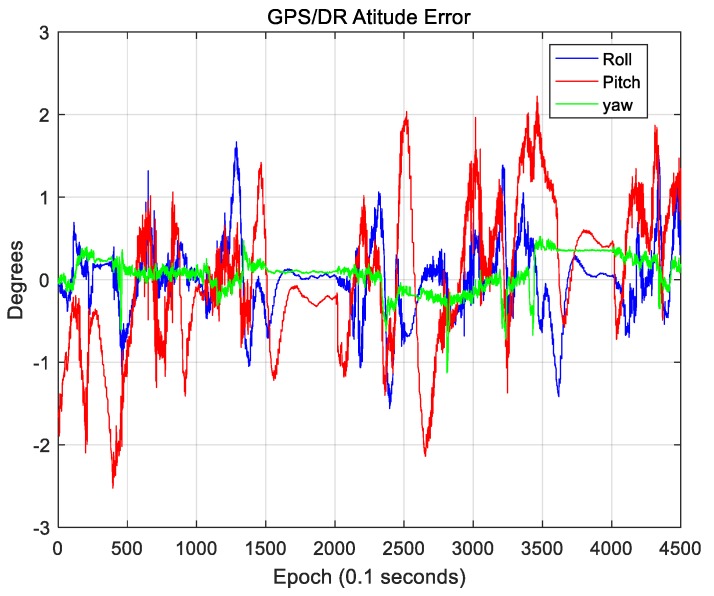
Attitude error of GPS/DR.

In this paper, the position error of the GPS/DR sensor was estimated using the map-matching results. For the GPS/DR sensor, a CruizCore DS6200 from Microinfinity (Suwon, South Korea) was used, and the azimuth accuracy was within 5° in open space. Figure 23 and Figure 24 show the position error and the attitude error of the GPS/DR sensor, respectively.

The position error was calculated based on the ground truth mentioned in Section 2.1, and the attitude error was calculated based on the roll, pitch, and yaw output values of the NovAtel RTK/SPAN System. Figure 23 shows that the position error of GPS/DR is up to 10 m. In addition, sections where the position error rapidly changes are mostly sections where the vehicle rotates. Such rapid changes in the error may lead to large position errors during the vehicle position estimation. 

As the position error of GPS/DR is very large in a downtown area, error correction through map matching is essential. In Figure 24, the roll and pitch errors were mostly within 2°, and the yaw error was mostly within 0.5°, indicating comparatively accurate results. Therefore, for the coordinate transformation of the 3D LIDAR data, the attitude information of GPS/DR was used as it was estimated separately, and the attitude of the vehicle was not separately estimated. 

The state variable of the filter can be represented as Equation (1):

(1) $$q={\left(\delta x\;,\;\;\delta y\right)}^{T}$$

where δx and δy refer to the 2D horizontal position errors of a vehicle in the East-North-Up (ENU) coordinate system. The time update is conducted as presented in Equation (2):

(2) $$\begin{array}{l} {\tilde{q}}_{k}=F_{K}\cdot {\hat{q}}_{k-1}\\ F_{k}=\left[\begin{array}{cc} 1 & 0\\ 0 & 1 \end{array}\right] \end{array}$$

As represented in Equation (2), no change in the error of GPS/DR for a short period of time was assumed. The measurement Equation is represented as Equation (3):

(3) $$\begin{array}{l} z_{k}=H_{k}\cdot {\tilde{q}}_{k}+\left[\begin{array}{c} \Delta x\\ \Delta y \end{array}\right]\\ H_{k}=\left[\begin{array}{cc} 1 & 0\\ 0 & 1 \end{array}\right] \end{array}$$

where Δx and Δy refer to the result values of the correlation-based matching. The measurement update is conducted as presented in Equation (4):

(4) $${\hat{q}}_{k}={\tilde{q}}_{k}+K(z_{k}-H\cdot {\tilde{q}}_{k})$$

where K refers to the Kalman gain.

## 4. Experimental Results

In this paper, localization was performed for three cases: a case in which only road markings are used, a case in which only vertical structures are used, and a case in which both road markings and vertical structures are used (ELM).

**Figure 25:**
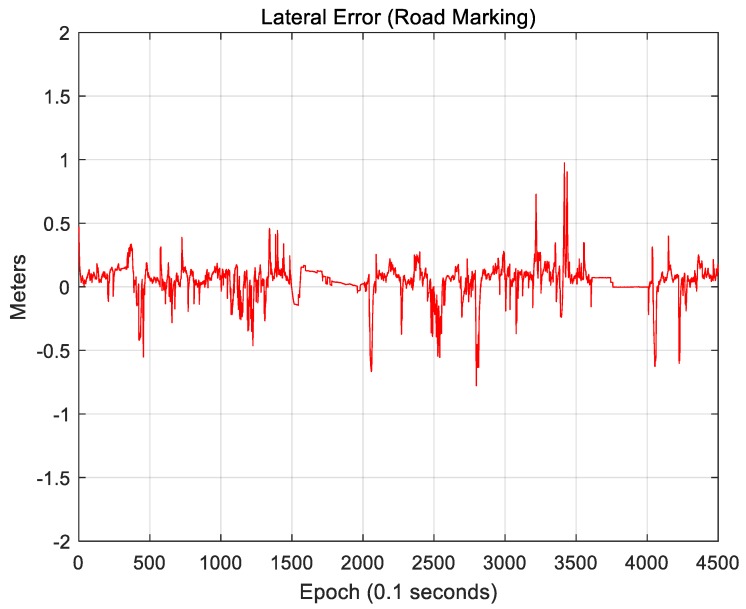
Lateral position error (road marking).

**Figure 26:**
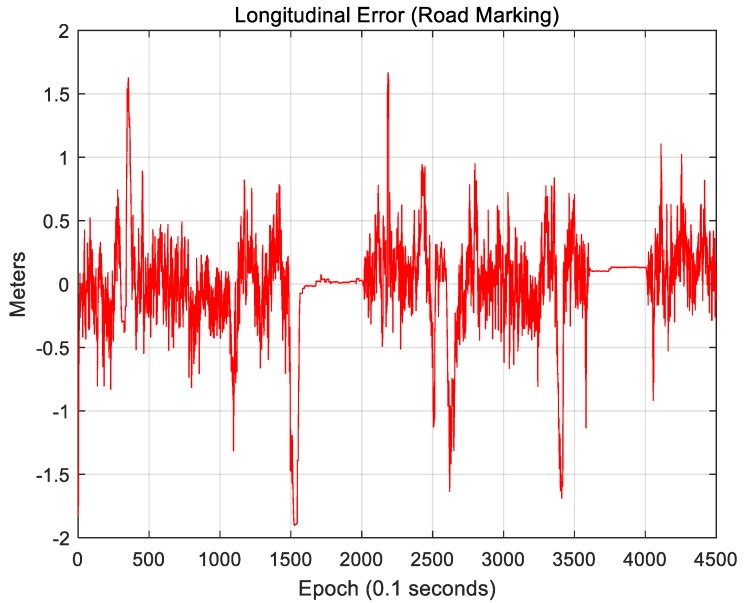
Longitudinal position error (road marking).

Figure 25 and Figure 26 show the localization results of the case in which only road markings were used.

Figure 25 shows the lateral position error. The RMS lateral position error is 0.143 m. Figure 26 shows the longitudinal position error. The RMS longitudinal position error is 0.389 m. When only road markings were used, the longitudinal position error was much larger than the lateral position error. As a road must have lanes, the lateral accuracy is high. To correct the longitudinal position error, certain road markings, such as crosswalks or stop lines, are necessary. On a number of roads, however, road markings other than lanes do not exist. Therefore, the longitudinal position error is somewhat higher.

**Figure 27:**
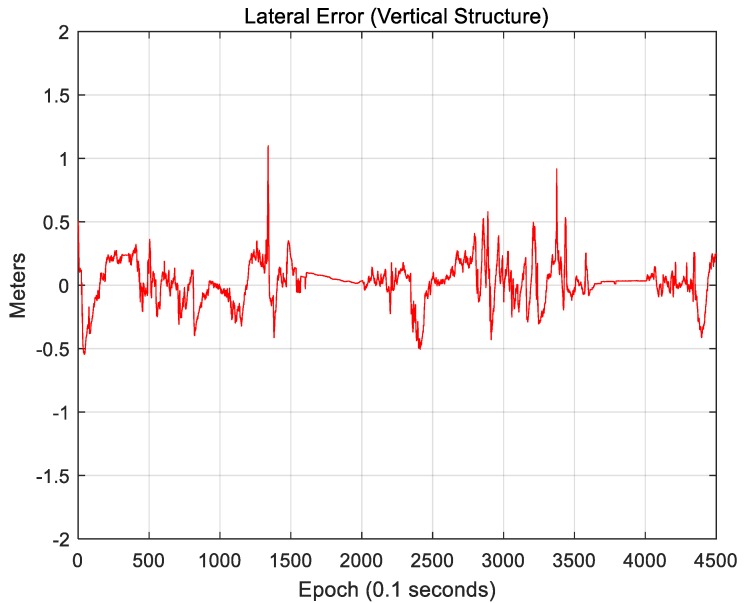
Lateral position error (vertical structure).

**Figure 28:**
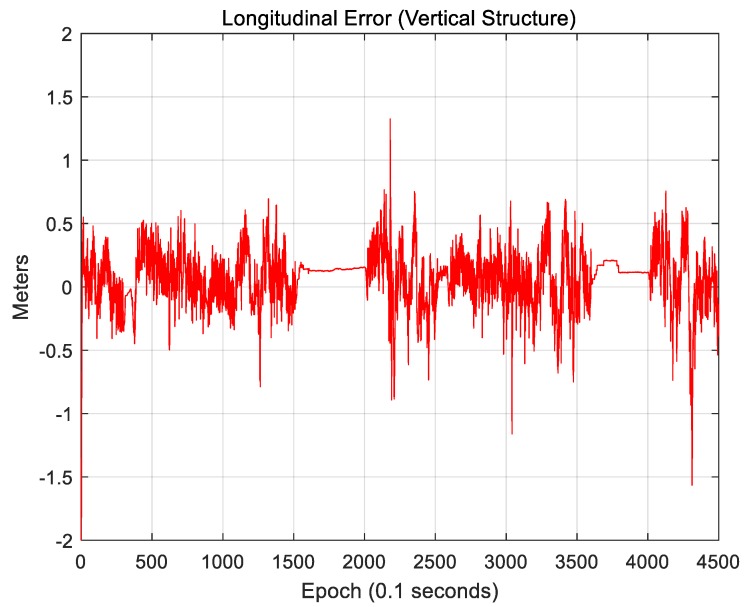
Longitudinal position error (vertical structure).

Figure 27 and Figure 28 show the localization results when only vertical structures were used.

Figure 27 shows the lateral position error. The RMS lateral position error is 0.163 m. Figure 28 shows the longitudinal position error. The RMS longitudinal position error is 0.233 m. When only vertical structures were used, it was found that the longitudinal position error significantly decreased. This is because building walls and traffic signs perpendicular to the vehicle traveling direction can provide measurements for the longitudinal direction. 

Likewise, the lateral position accuracy is high because there are buildings on both sides of the road. Vertical structures provide highly reliable measurements in a downtown area. However, as they are typically tens of meters away from the vehicle, the localization accuracy is significantly affected by the roll and pitch errors. As road markings are available closer to the vehicle, the localization accuracy in an area with a large number of road markings will be higher when the road markings are used than when vertical structures are used. Actually, as shown in Figure 25, although large position errors occasionally occur, the RMS position error is smaller than that when only vertical structures are used. 

**Figure 29:**
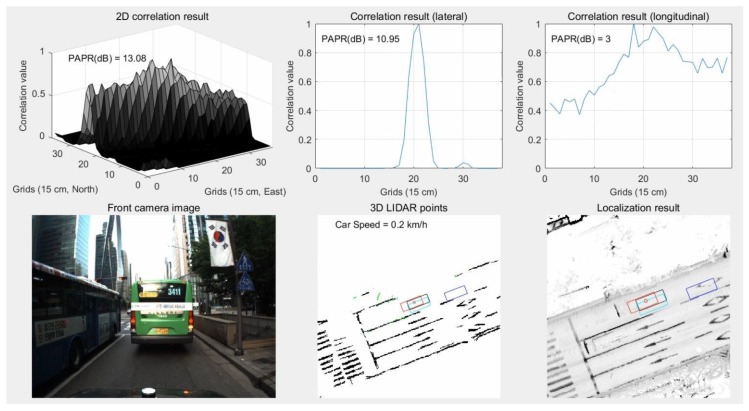
Results of localization using road marking in area with traffic congestion.

It is difficult to use road markings for localization in areas with traffic congestion. Figure 29 shows the results of localization using road markings in an area with traffic congestion.

**Figure 30:**
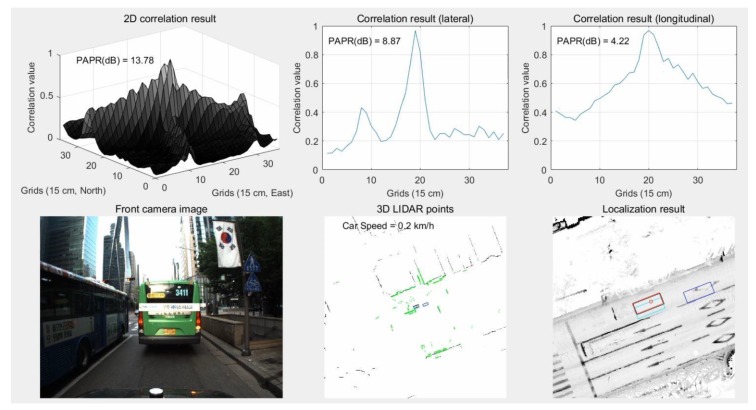
Results of localization using vertical structures in area with traffic congestion.

As can be seen from the camera image of Figure 29, most road markings are hidden by surrounding vehicles. In the bottom center of the figure, road markings (light green dots) that can be matched to the map were not significantly detected. As shown at the top of the figure, lateral matching is well-performed owing to some lanes and curbs, but longitudinal matching is not performed well. As a result, in the bottom-right hand figure, the estimated vehicle position (red) has a large error in the longitudinal direction compared to the ground truth (black). On the other hand, in the same area, vertical structures can be scanned without the influence of surrounding vehicles. Figure 30 shows the results of localization using vertical structures in an area with traffic congestion.

As can be seen from Figure 30, longitudinal matching is performed well even in an area with traffic congestion. As a result, the bottom-right hand figure shows that the red rectangle overlaps the black one. 

**Figure 31:**
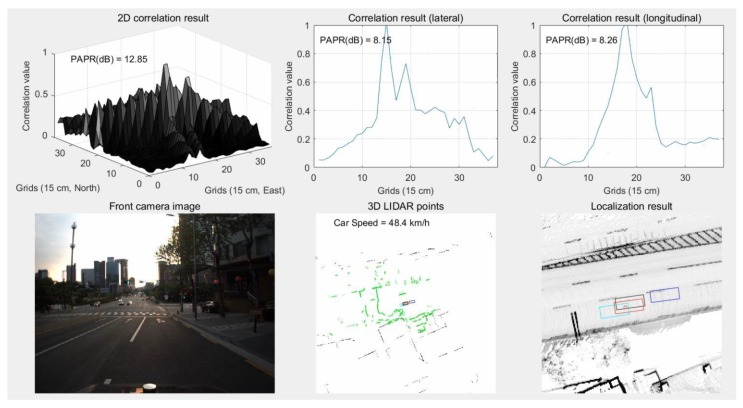
Results of localization using vertical structures in area with a small number of nearby buildings.

In contrast to the area with traffic congestion, in an area with a small number of nearby buildings, the performance of localization using vertical structures can be degraded. Figure 31 shows the results of localization using vertical structures in an area with a small number of nearby buildings.

**Figure 32:**
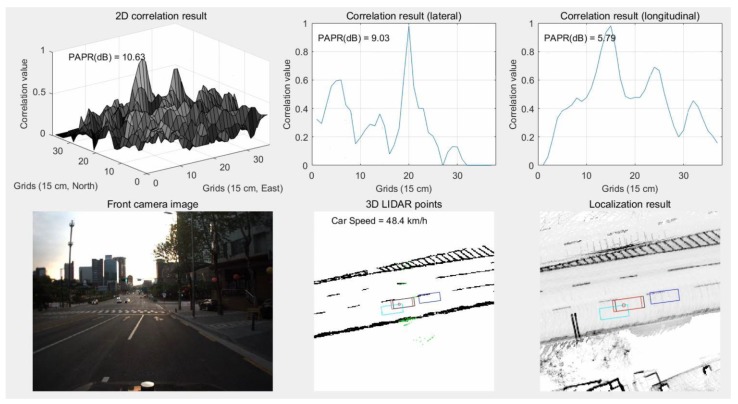
Results of localization using road markings in an area with a small number of nearby buildings.

As can be seen from the camera image of Figure 31, there are not many buildings on the left side of the road. In this area, multiple peaks appear in the correlation shape for the lateral direction. Furthermore, the side peak is larger than the main peak. As a result, as shown in the bottom-right hand figure, the red rectangle has a lateral position error compared to the black one. On the other hand, if road markings are used in the same area, the lateral position error can be reduced. Figure 32 shows the results of localization using road markings in an area with a small number of nearby buildings.

As shown in Figure 32, the main peak can be clearly detected in the correlation shape for the lateral direction. As a result, it can be seen that the estimated vehicle position has almost no lateral error.

**Figure 33:**
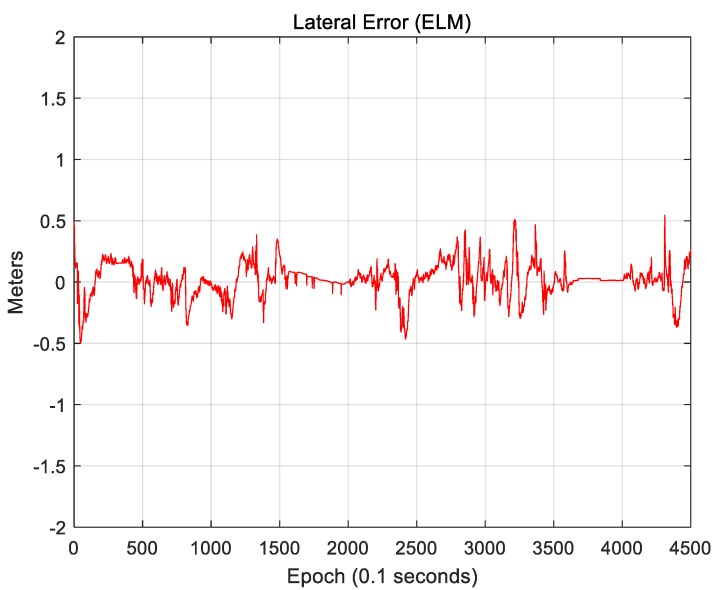
Lateral position error (ELM).

**Figure 34:**
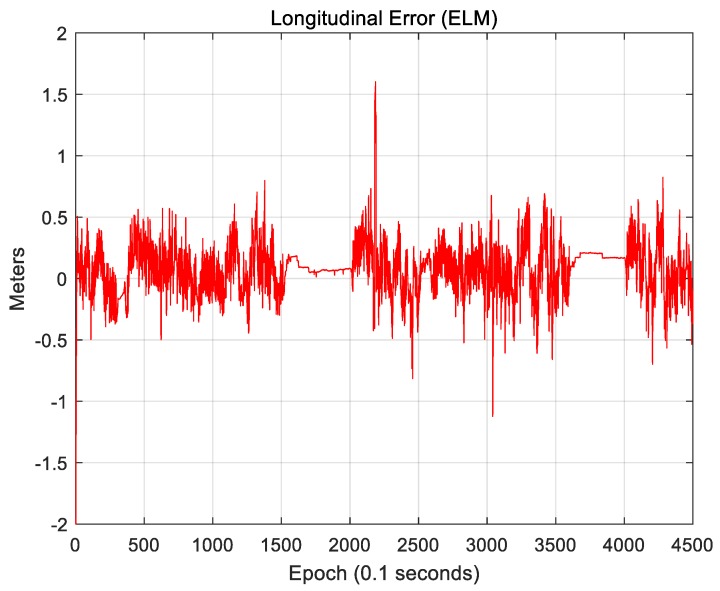
Longitudinal position error (ELM).

As seen, road markings and vertical structures can supplement each other. Therefore, the use of an ELM, which includes both attributes, can exhibit further improved localization accuracy. Figure 33 and Figure 34 show the results of localization using an ELM (road markings + vertical structures).

Figure 33 shows the lateral position error. The RMS lateral position error is 0.136 m. Figure 34 shows the longitudinal position error. The RMS longitudinal position error is 0.223 m. Figure 33 and Figure 34 show that both the lateral and longitudinal position errors were reduced. In particular, the number of sections with large position errors was significantly reduced. Table 2 lists the localization performances of the three maps (road markings, vertical structures, and ELM).

Table 2 shows that the use of an ELM exhibited the smallest RMS position errors in both the lateral and longitudinal directions. At a 95% confidence level, road markings caused the smallest lateral position error, and the ELM led to the smallest longitudinal position error. At a 99% confidence level, the ELM exhibited the smallest lateral and longitudinal position errors. As seen, ELM-based localization shows higher position accuracy than the use of either road markings or vertical structures. This is because the amount of map information available at the time of localization is increased owing to the mutual complement of road markings and vertical structures.

The localization accuracy improves as the amount of map information increases. When the ELM is converted into an occupancy grid map, this map is filled with only 0 and 1. In this case, if 0 and 1 are seen as the elevation of the terrain, the concept of terrain roughness can be used to determine the amount of map information [29,30,31,32]. The terrain roughness can be expressed using the two indices of sigma-T and sigma-Z, as follows:

(5) $$\sigma_{T}=\sqrt{\frac{1}{N}\sum_{i=1}^{N}{\left(H_{i}-\bar{H}\right)}^{2}}\;\;\;\;\;\;\;\;\;\;\;\;\bar{H}=\frac{1}{N}\sum_{i=1}^{N}H_{i}$$

(6) $$\sigma_{Z}=\sqrt{\frac{1}{N-1}\sum_{i=1}^{N-1}{\left(D_{i}-\bar{D}\right)}^{2}}\;\;\;\;\;\;\;\;\;\;\;\;D_{i}=H_{i+1}-H_{i}\;\;\;\;\;\;\;\;\;\;\;\;\bar{D}=\frac{1}{N-1}\sum_{i=1}^{N-1}D_{i}$$

In Equation (5), $H_{i}$ is the elevation information of the *i*-th grid, and N is the number of grids in the map. Sigma-T is the standard deviation of the elevation, and sigma-Z is the standard deviation of the elevation difference of neighboring grids. In general, the localization accuracy is higher as the values of sigma-T and sigma-Z become higher.

**Figure 35:**
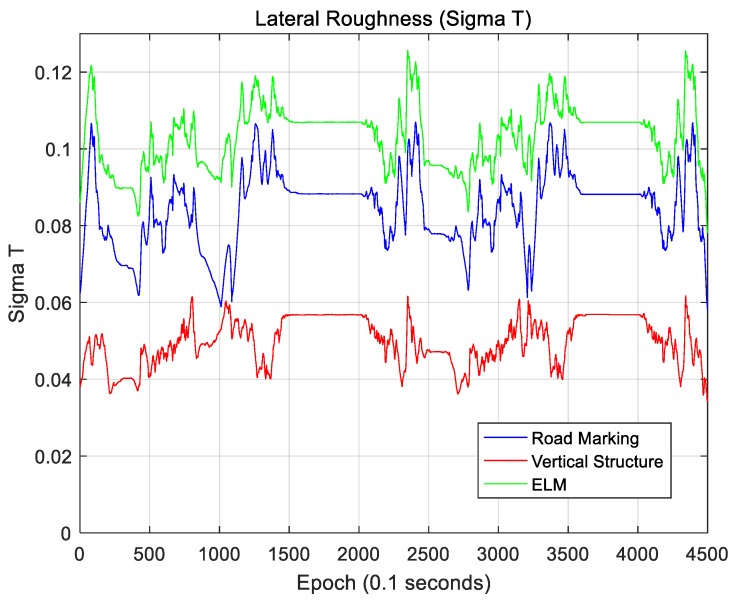
Lateral roughness (Sigma-T).

**Figure 36:**
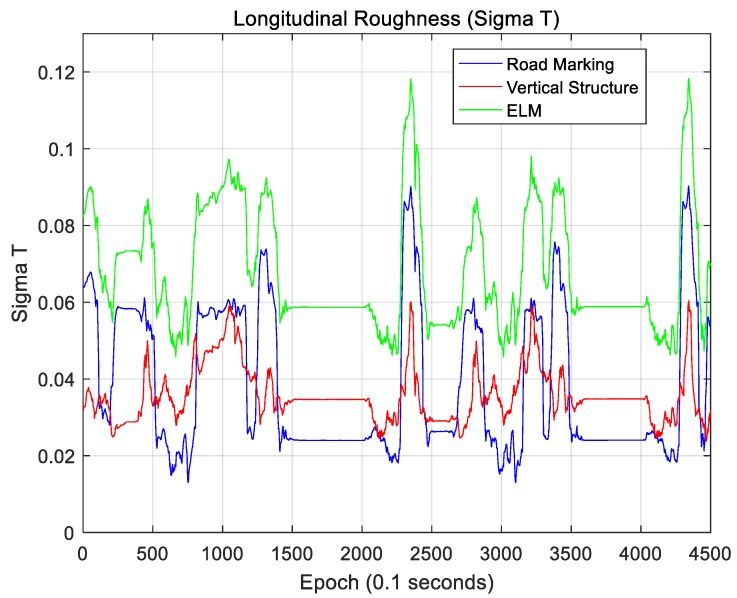
Longitudinal roughness (Sigma-T).

**Figure 37:**
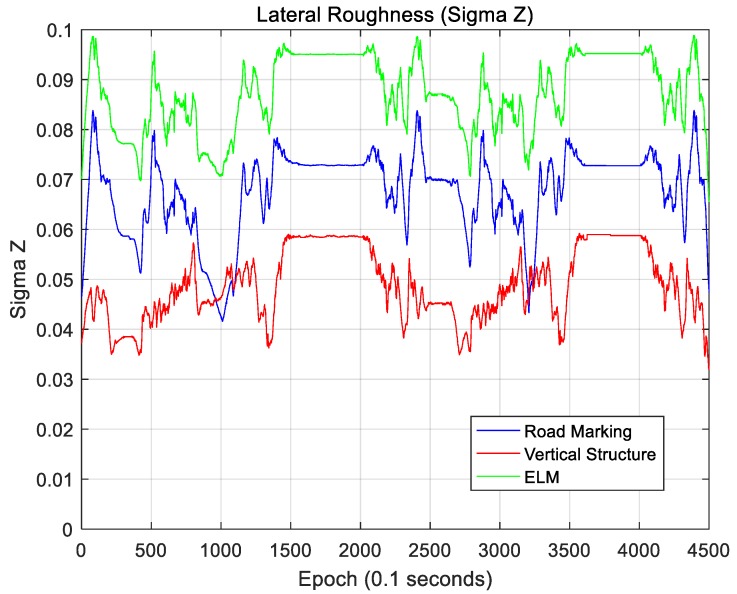
Lateral roughness (Sigma-Z).

**Figure 38:**
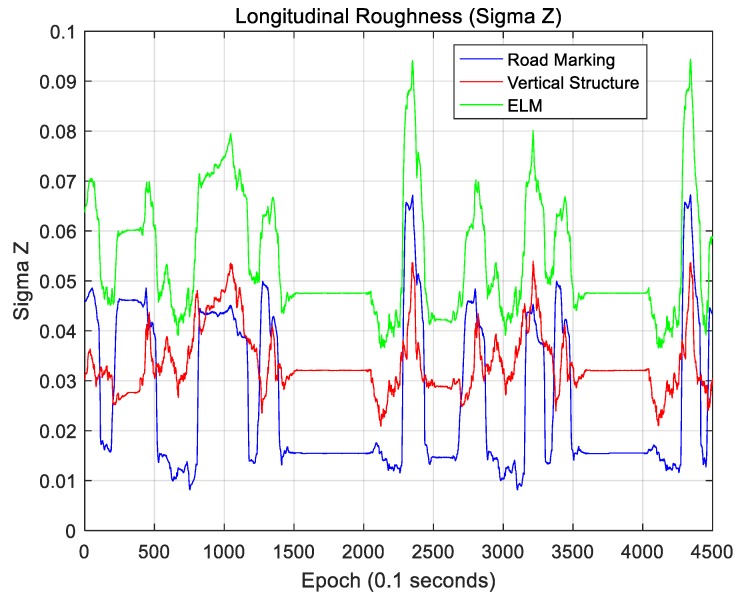
Longitudinal roughness (Sigma-Z).

The ELM is expressed as a 2D occupancy grid map. Therefore, sigma-T and sigma-Z must be calculated on the 2D plane. In this paper, sigma-T and sigma-Z are calculated as follows:

(7) $$\begin{array}{l} \sigma_{T,Lateral}=\frac{1}{N}\sum_{i=1}^{N}\sqrt{\frac{1}{N}\sum_{j=1}^{N}{\left(H_{ij}-{\bar{H}}_{i}\right)}^{2}}\;\;\;\;\;\;\;\;\;\;\;\;{\bar{H}}_{i}=\frac{1}{N}\sum_{j=1}^{N}H_{ij}\\ \sigma_{T,Longitudinal}=\frac{1}{N}\sum_{j=1}^{N}\sqrt{\frac{1}{N}\sum_{i=1}^{N}{\left(H_{ij}-{\bar{H}}_{j}\right)}^{2}}\;\;\;\;\;\;\;\;\;\;\;\;{\bar{H}}_{j}=\frac{1}{N}\sum_{i=1}^{N}H_{ij} \end{array}$$

(8) $$\begin{array}{l} \sigma_{Z,Lateral}=\frac{1}{N}\sum_{i=1}^{N}\sqrt{\frac{1}{N-1}\sum_{j=1}^{N-1}{\left(D_{ij,Lat}-{\bar{D}}_{i}\right)}^{2}}\;\;\;\;\;\;\;\;\;\;\;\;D_{ij,Lat}=H_{i+1,j}-H_{ij}\;\;\;\;\;\;\;\;\;\;\;\;{\bar{D}}_{i}=\frac{1}{N-1}\sum_{i=1}^{N-1}D_{ij,Lat}\\ \sigma_{Z,Longitudinal}=\frac{1}{N}\sum_{j=1}^{N}\sqrt{\frac{1}{N-1}\sum_{i=1}^{N-1}{\left(D_{ij,Long}-{\bar{D}}_{j}\right)}^{2}}\;\;\;\;\;\;\;\;\;\;\;\;D_{ij,Long}=H_{i,j+1}-H_{ij}\;\;\;\;\;\;\;\;\;\;\;\;{\bar{D}}_{j}=\frac{1}{N-1}\sum_{j=1}^{N-1}D_{ij,Long} \end{array}$$

where N is the map size in the area of interest based on the current vehicle position, and this map is a N×N matrix. For a localization performance analysis, this map is rotated using the current vehicle azimuth information so that the longitudinal direction faces north. Therefore, $H_{ij}$ denotes the elevation of the grid located at the *i*-th position in the longitudinal direction and the *j*-th position in the lateral direction on the rotated map. Figure 35 and Figure 36 show sigma-T for the lateral and longitudinal directions, respectively. Figure 37 and Figure 38 show the sigma-Z for the lateral and longitudinal directions, respectively.

Sigma-T is related to the magnitude of the correlation peak, while sigma-Z is related to the sharpness of the main peak. For this reason, both sigma-T and sigma-Z must be large to find the correlation peak more accurately. The above figures show that the roughness of the ELM is the highest. Furthermore, the lateral roughness generally has higher values than the longitudinal roughness. As a result, the lateral accuracy is higher than the longitudinal accuracy, as shown in Table 2. In addition, when the RMS position errors for road markings, vertical structures, and the ELM were compared with the roughness values, it was found that as the roughness increased, the RMS position error decreased. In Figure 34, Figure 36, and Figure 38, the points at which the longitudinal position error was 0.5 m or more also show low longitudinal roughness values. 

As seen, the ELM has the highest roughness as well as the most excellent localization performance. Although the ELM has a very small data file size (approximately 134 KB/km), it has sufficient information required for accurate localization. As it contains both road markings and vertical structure information, it is possible to continuously perform localization even in areas with traffic congestion or in areas without surrounding buildings. Furthermore, the ELM-based localization method sufficiently meets the localization accuracy requirements for autonomous driving.

## 5. Conclusions

This paper proposed an ELM-based precise vehicle localization method using 3D LIDAR. The proposed ELM has a very small data file size (134 KB/km). Furthermore, as it contains both road markings and vertical structure information, the ELM-based localization method exhibits better performance in terms of accuracy, reliability, and availability than methods that use either road markings or vertical structures. In addition, ELM can be generated through the ELM generation algorithm, and no verification is required for the detected lines. Furthermore, as ELM has a form that is extremely similar to the actually produced line maps of lanes, ELM is easily compatible with similar maps.

As a result of ELM-based localization, the RMS position errors for the lateral and longitudinal directions were 0.136 m and 0.223 m, respectively. These results sufficiently met the localization accuracy requirements for autonomous driving. In addition, line detection and data association processes are not required because the correlation matching method was used. Furthermore, correlation matching can be performed using a Fast Fourier Transform (FFT) because simple multiplication for 0 and 1 is required. The matching time using FFT is approximately 78 ms.

As seen, the ELM-based localization method can ensure high position accuracy using a map with a small data size. In the future, research on map generation and localization in more areas is required. 

## Figures and Tables

**Table 1:** Example of ELM.

Link Index	Type	Node 1		Node 2	
		Latitude	Longitude	Latitude	Longitude
1	Road Marking	37.5116372276	127.0574300149	37.5116732539	127.0574065740
2	Building	37.5124894367	127.0579568743	37.5125936427	127.0578932581

**Table 2:** Localization performances of three maps.

Type of Map	Position Error (m)					
	RMS		95% Confidence Level		99% Confidence Level	
	Lateral	Longitudinal	Lateral	Longitudinal	Lateral	Longitudinal
Road Marking	0.143	0.389	0.27	0.82	0.56	1.59
Vertical Structure	0.163	0.233	0.34	0.45	0.49	0.67
ELM	0.136	0.223	0.29	0.42	0.42	0.60
